# [Corrigendum] MicroRNA‑23a inhibits endometrial cancer cell development by targeting SIX1

**DOI:** 10.3892/ol.2023.14032

**Published:** 2023-08-29

**Authors:** Hong-Lin Li, Jun-Jie Sun, Hui Ma, Shen-Jia Liu, Na Li, Su-Jie Guo, Yang Shi, Yan-Ying Xu, Zhi-Ying Qi, Yu-Quan Wang, Fang Wang, Rui-Meng Guo, Dong Liu, Feng-Xia Xue

Oncol Lett 18: 3792–3802, 2019; DOI: 10.3892/ol.2019.10694

Following the publication of the above article, an interested reader drew to the authors’ attention that, in Fig. 4D on p. 3797 showing the results of Transwell invasion assay experiments, sections of the ‘HEC1B/SIX1 con’ and ‘Ishikawa/SIX1 treated’ data panels were overlapping, such that these data appeared to have been derived from the same original source where the results of differently performed experiments were intended to have been shown. After having re-examined their original data, the authors realized that this figure had inadvertently been assembled incorrectly.

A revised version of Fig. 4, showing data from one of the repeated set of experiments for Fig. 4D, is shown on the next page. Note that the errors made in assembling this figure did not affect the overall conclusions reported in the paper. All the authors agree to the publication of this corrigendum, and they are grateful to the Editor of *Oncology Letters* for allowing them the opportunity to publish this. The authors also apologize to the readership for any inconvenience caused.

## Figures and Tables

**Figure 4.**
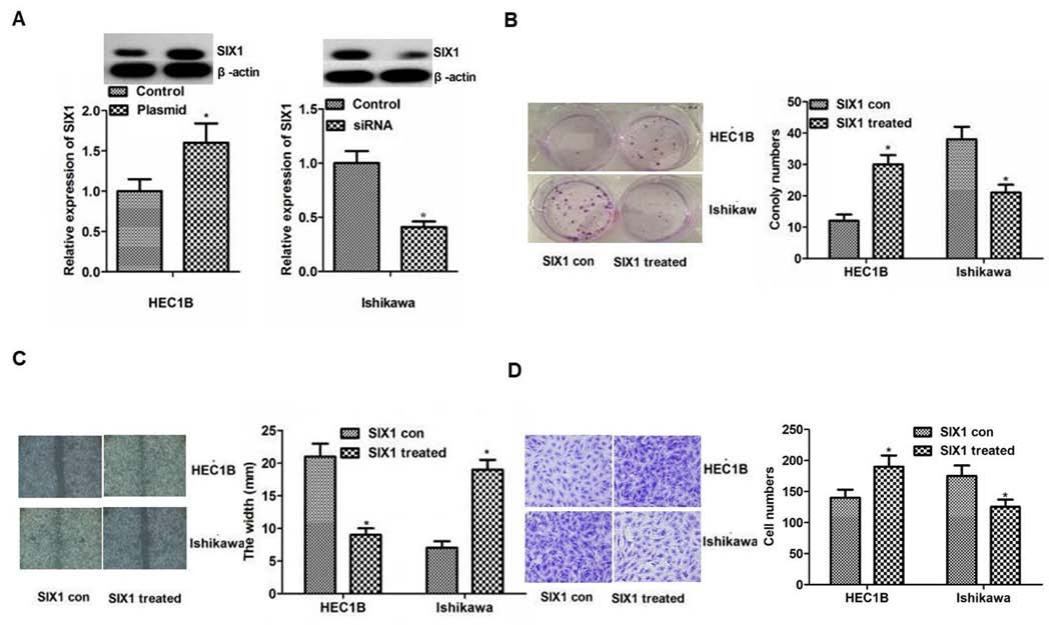
Influence of SIX1 on the biological behavior of endometrial cancer cell lines. (A) SIX1 plasmid or siRNA were used to increase or reduce the protein expression levels of SIX1 in HEC1B or Ishikawa cells. Differences were detected using western blotting. Protein expression levels of SIX1 in HEC1B or Ishikawa cells were increased or reduced in the SIX1 plasmid or siRNA group compared with the control group, respectively. (B) Colony formation assay. The results of colony formation assay demonstrated that proliferation was significantly enhanced or weakened compared with the control in HEC1B or Ishikawa cells, respectively. Magnification, ×200. (C) Migration detected by wound healing assays. The results revealed significantly smaller or bigger width in the treated group compared with the control group in HEC1B or Ishikawa cells, respectively. (D) Invasion detected by Transwell assays. Significantly more or fewer invasive cells were observed in the treated group compared with in the control group in HEC1B or Ishikawa cells, respectively. Magnification, ×200. *P<0.05 vs. con. Con, control; siRNA, small interfering RNA; SIX1, sine oculis homeobox homolog 1; SIX1 treated, overexpression in the HEC1B cell line or knockdown in the Ishikawa cell line.

